# Cell array-based intracellular localization screening reveals novel functional features of human chromosome 21 proteins

**DOI:** 10.1186/1471-2164-7-155

**Published:** 2006-06-16

**Authors:** Yu-Hui Hu, Hans-Jörg Warnatz, Dominique Vanhecke, Florian Wagner, Andrea Fiebitz, Sabine Thamm, Pascal Kahlem, Hans Lehrach, Marie-Laure Yaspo, Michal Janitz

**Affiliations:** 1 Department of Vertebrate Genomics, Max Planck Institute for Molecular Genetics, 14195 Berlin, Germany; 2 FU Berlin, Department of Biology, Chemistry and Pharmacy, 14195 Berlin, Germany; 3 RZPD German Resource Center for Genome Research, 14059 Berlin, Germany; 4 Department of Hematology, Oncology, and Tumor Immunology, Humboldt University, Charite, Berlin, Germany

## Abstract

**Background:**

Trisomy of human chromosome 21 (Chr21) results in Down's syndrome, a complex developmental and neurodegenerative disease. Molecular analysis of Down's syndrome, however, poses a particular challenge, because the aneuploid region of Chr21 contains many genes of unknown function. Subcellular localization of human Chr21 proteins may contribute to further understanding of the functions and regulatory mechanisms of the genes that code for these proteins. Following this idea, we used a transfected-cell array technique to perform a rapid and cost-effective analysis of the intracellular distribution of Chr 21 proteins.

**Results:**

We chose 89 genes that were distributed over the majority of 21q, ranging from RBM11 (14.5 Mb) to MCM3AP (46.6 Mb), with part of them expressed aberrantly in the Down's syndrome mouse model. Open reading frames of these genes were cloned into a mammalian expression vector with an amino-terminal His_6 _tag. All of the constructs were arrayed on glass slides and reverse transfected into HEK293T cells for protein expression. Co-localization detection using a set of organelle markers was carried out for each Chr21 protein. Here, we report the subcellular localization properties of 52 proteins. For 34 of these proteins, their localization is described for the first time. Furthermore, the alteration in cell morphology and growth as a result of protein over-expression for claudin-8 and claudin-14 genes has been characterized.

**Conclusion:**

The cell array-based protein expression and detection approach is a cost-effective platform for large-scale functional analyses, including protein subcellular localization and cell phenotype screening. The results from this study reveal novel functional features of human Chr21 proteins, which should contribute to further understanding of the molecular pathology of Down's syndrome.

## Background

Eukaryotic cells are characterized by a high degree of compartmentalization and most protein activities can be assigned to particular cellular compartments. Moreover, the accurate functions of protein interaction networks rely greatly on the proper localization of each protein component. In many cases, aberrant translocation of proteins correlates highly with pathological changes in cell physiology. Until recently, protein localization experiments have been confined to only one or a few particular genes of interest. The first large-scale localization study in mammalian cells was performed in microwell-plate format by Simpson *et al*. and included 107 human genes [1]. Recently, an automated transfection and immunostaining system for 96-well plates has been established [2]. A microwell-plate-based approach, however, is associated with a high consumption of reagents and a requirement for extensive automation. A recently developed transfected cell array (TCA) technique [3] represents a cost-effective alternative for high-throughput approaches in functional genomics. The principle of the TCA technique is based on the transfection of DNA or RNA molecules, immobilized on a solid surface, into mammalian cells. Subsequently, detection of the physiological effects on these cells caused by the introduction of foreign nucleic acid is carried out [4]. We optimized this platform for high-throughput protein co-localization studies and applied it to the characterization of human chromosome 21 (Chr21) encoded proteins.

**Figure 1:**
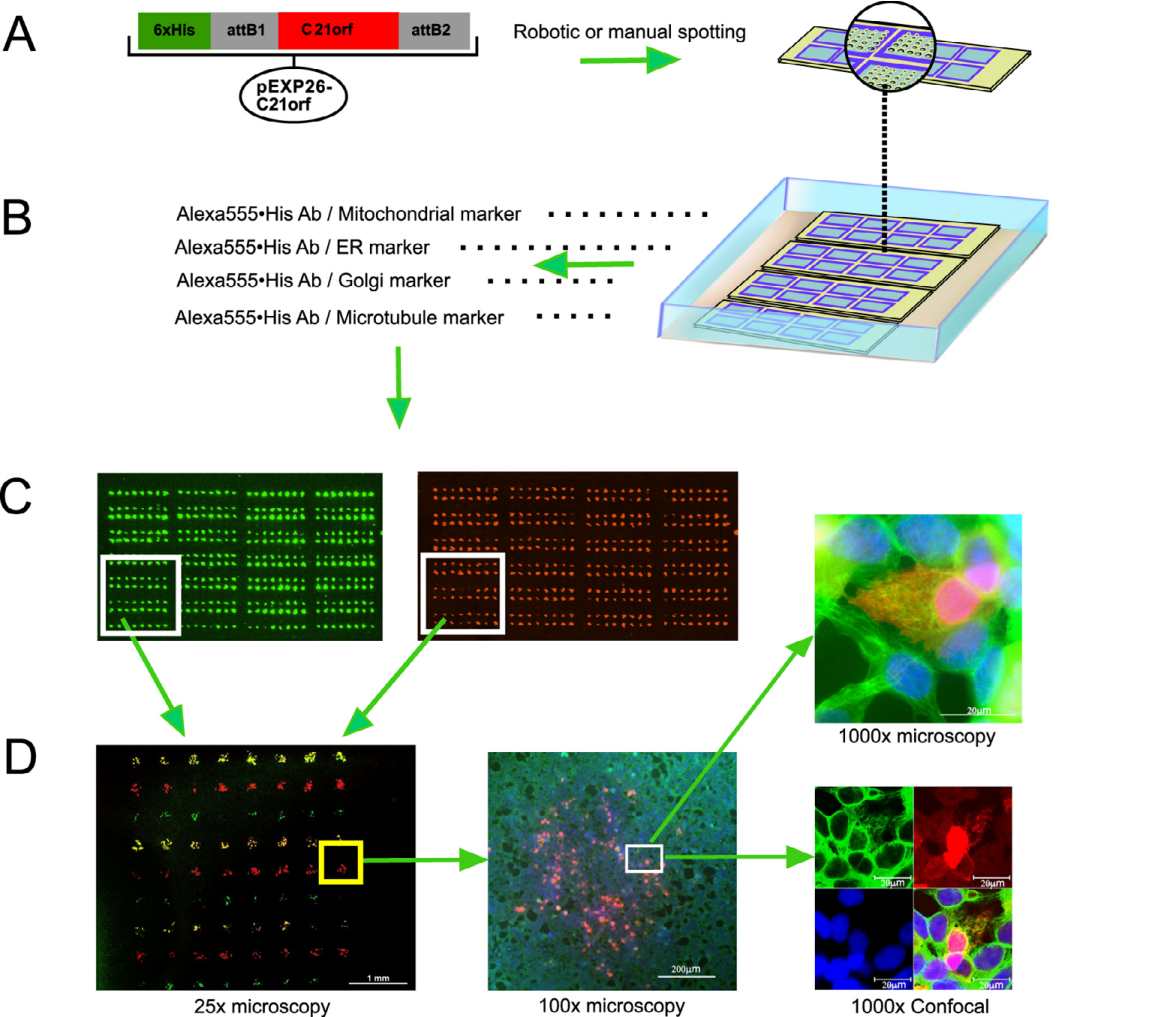
Schematic presentation of the cell array-based protein localization procedure. (A) Open reading frames (ORFs) derived from human chromosome 21 were cloned into a mammalian expression vector containing an amino-terminal His_6 _tag, spotted in an array format and reverse transfected into an HEK293T cell line. (B) Proteins expressed on the cell arrays were detected using anti-His antibody and a set of organelle counterstaining was performed. (C) The efficiency of protein expression and labeling was monitored using a BioCCD laser scanning system. The green signal indicates the expression of enhanced green fluorescent protein (EGFP). The red signal indicates His_6_-tagged protein. (D) For single-cell analysis, fluorescent and confocal microscopy was used. The red signal in the 1000× magnification images represents expressed C21orf25 protein, whereas the green and blue signals represent labeled microtubule and nucleus, respectively.

Functional analysis of Chr21 proteins is of great medical relevance. This refers, in particular, to trisomy of human Chr21, which results in Down's syndrome, a complex developmental and neurodegenerative disease. The phenotype of Down's syndrome includes various organ malformations, stereotypic craniofacial anomalies and brain malformations [5]. Molecular analysis of this syndrome, however, poses a particular challenge, because the aneuploid region of Chr21 contains genes of unknown function. Genomic sequencing and gene expression analysis of human Chr21 [6-10], as well as study of the transcriptome of a Down's syndrome mouse models [11-15], provide a comprehensive resource for the systematic functional characterization of Chr21 genes. However, functional characterization at the protein level has been performed most often using protein prediction algorithms. Therefore, determination of the subcellular protein distribution would provide an important insight into the function of human Chr21 genes. In the present study, 89 human Chr21 genes were analyzed for subcellular localization of protein using a TCA technique (Fig. 1). Furthermore, changes in cellular phenotypes, such as cell morphology and proliferation, could be identified as a consequence of over-expression of some of these genes.

## Results and discussion

### Subcellular localization of Chr21 proteins

In order to ensure that the localization of the expressed proteins has not been influenced by the cell array protocol or His_6 _tagging, genes coding for proteins with well-described localization to particular cellular compartments were reverse transfected and over-expressed. The control genes encoded for the CDH8, KDELR1, LMNA, Pex11a, TGN38 and EGFP proteins [16-21]. All control proteins have been detected in the locations as described [22].

The subcellular localization pattern for 52 out of 89 Chr21 proteins was determined using His_6 _epitope detection. The rest of the proteins could not be detected, as their expression remains below the detection level of the cell array. The subcellular distribution for mammalian cells of 34 detected proteins is described here for the first time. A summary of the localization data is presented in Table 1 [see Additional file 1], together with the protein and gene accession numbers, and information on gene function and expression patterns. Localization images can be visualized and downloaded from [22]. In order to exclude the possibility that the fusion of the His_6 _tag at the protein N terminus influenced its localization, the Chr21 open reading frames (ORFs) have been cloned with a Myc epitope tag at the C terminus. The localization of the C terminus-tagged proteins did not substantially differed from the localization pattern of N terminus-tagged proteins (data not shown).

**Figure 2:**
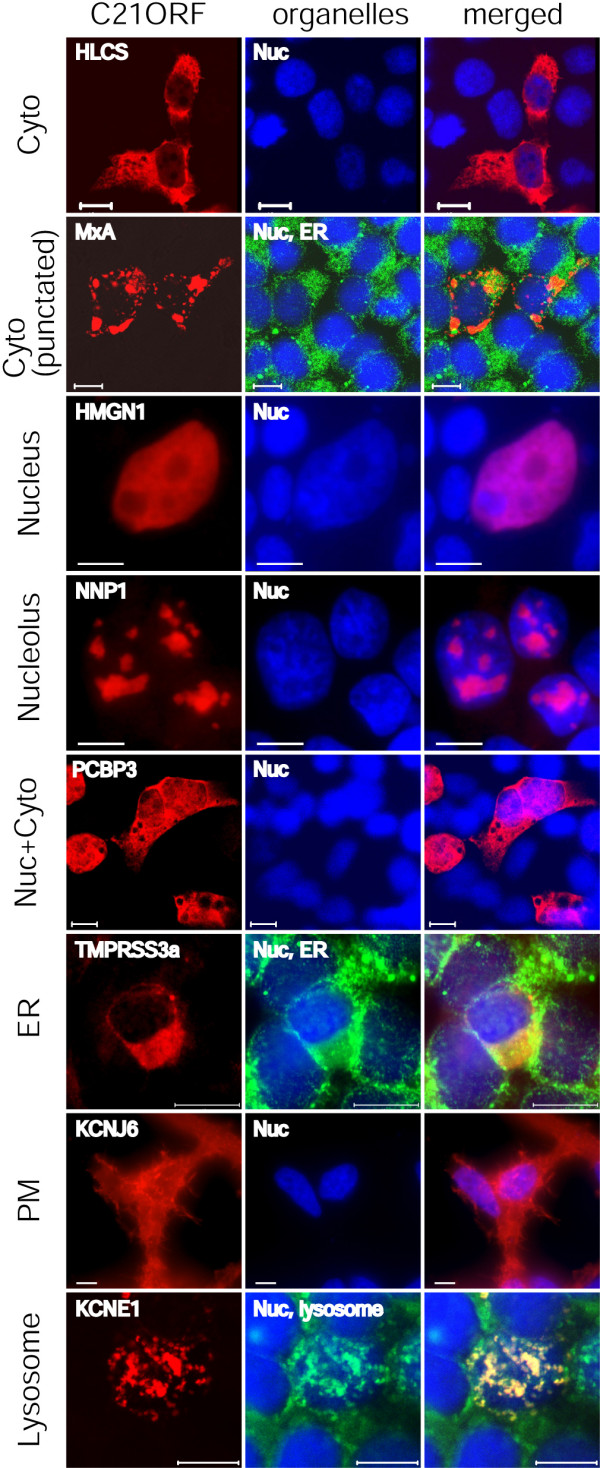
Localization categories for detected chromosome 21 (Chr21) proteins. The Chr21 proteins were localized in a variety of cellular compartments. The images in the left panel represent anti-His staining of Chr21 proteins, with the gene names indicated in the upper left corner of each image. The images in the middle panel show applied counterstaining, with organelles indicated in the upper left corner. The images in the right panel present a merged picture from detection of Ch21 proteins and counterstaining. The scale bar represents 10 μm.

A significant proportion of the proteins localized either in the cytosol (31%) or in the nucleus (17%). In all, 23% of proteins were found in both the cytoplasm and the nucleus, or were found to translocate between these two compartments. Localization of 29% of the proteins was associated with components of the endoplasmic reticulum (ER) secretory pathway, including the ER (10%), the Golgi (5%) and endosome/lysosome compartment (4%); 10% of proteins were localized in the plasma membrane (PM). Figure 2 shows localization examples of Chr21 proteins in different cellular compartments. In what follows, several features of protein distribution are discussed.

#### Cytosol

Two distinct localization features were observed for cytosolic proteins. Proteins were either distributed evenly throughout the cytoplasm or formed a punctate pattern of distribution. For example, the *HLCS *gene codes for a biotin-protein ligase and was found to localize throughout the cytosol (Fig. 2). Another protein, myxovirus resistance protein 1, which is encoded by the *MxA *gene, revealed a punctate pattern in the cytosol, which does not overlap with any organelle staining. The accumulated localization pattern of MxA in our study supports previous suggestions that self-assembled MxA proteins wrap around or interact with the incoming viral nucleocapsids and thereby prevent their replication [23]. Accola *et al*. reported that the MxA protein localizes to the smooth ER and might inhibit viral replication through alterations in membrane organization [24]. We did not, however, observe the co-localization of MxA with the ER marker, protein disulphide isomerase (PDI) (Fig. 2).

#### Nucleus

The non-histone chromosomal protein HMG-14, which is encoded by the *HMGN1 *gene, was found in the nucleus but outside of the nucleolus (Fig. 2). This localization correlated with its suggested function as a modulator of the interaction between DNA and the histone octamer through the binding of HMG-14 to nucleosomal DNA. In contrast, the novel nuclear protein 1 (NNP-1) localized exclusively in the nucleolus, in accordance with its role in the synthesis of 28 S rRNA (Fig. 2) [25].

#### Nucleus and cytoplasm

**Figure 3:**
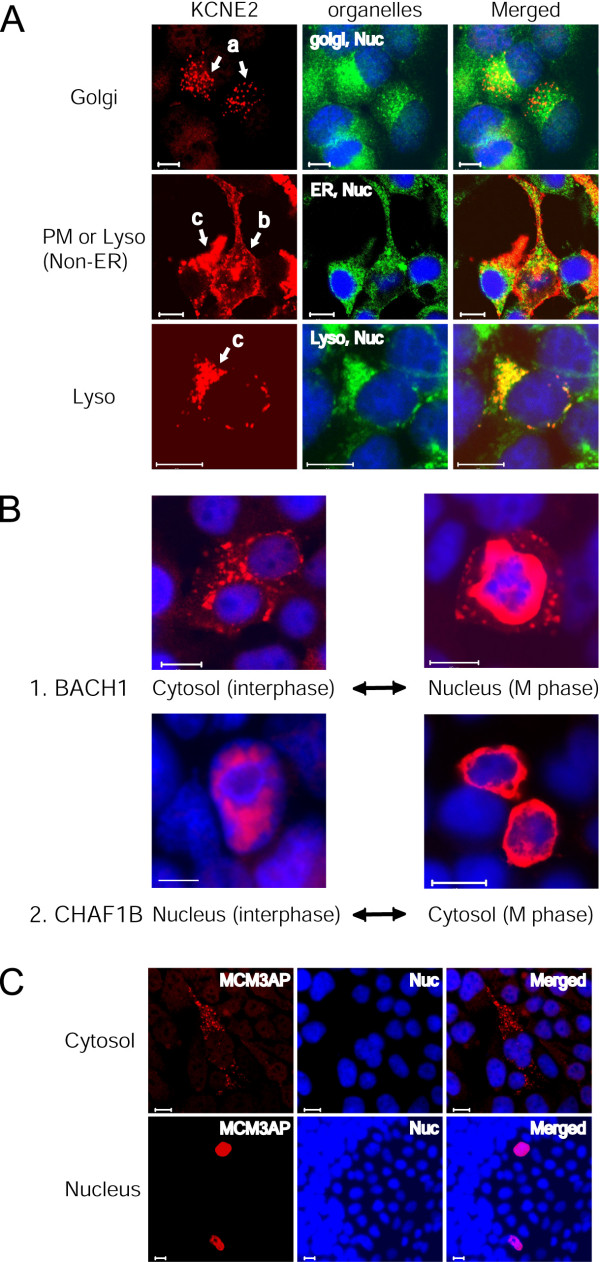
Protein intracellular translocation observed on cell arrays. (A) Stages of post-translational trafficking of KCNE2, a plasma membrane (PM) protein, including the early modification within the Golgi complex (a) and final localization at the PM (b) or in the lysosomes (c), without retention in the ER. (B) Translocation of BACH1 and CHAF1B proteins during cell interphase and mitosis. 1. BACH1 proteins distributed in the entire cytosol at interphase. In early mitosis, most proteins accumulated in the nucleus and wrapped around condensed chromosome. 2. During the interphase, CHAF1B protein localized in the nucleus, whereas after cell division it was found in the cytoplasm of two daughter cells. (C) Differential localization patterns of MCM3AP protein in the cytoplasm and nucleus. The scale bar represents 10 μm.

In all, 23% of Chr21 proteins were found to localize in both the nucleus and the cytoplasm. The nucleus/cytoplasm distribution ratio for a particular protein, however, varied from cell to cell. In some cells, the proteins with dual localization could be found either only in the nucleus or only in the cytoplasm, suggesting a continuous translocation activity. For example, the variable distribution of the MCM3AP protein (minichromosome maintenance protein 3-associated protein) in the nucleus and cytoplasm (Fig. 3C) correlates with its role in the translocation of MCM3 proteins from the cytosol into the nucleus. The MCM3 protein is known to be an essential factor that allows DNA to undergo a single round of replication per cell cycle [26,27]. The requirement for nuclear import of MCM3 protein changes according to the phase of the cell cycle, which is probably reflected by variable distribution of MCM3AP between the nucleus and the cytosol [26].

#### ER-associated secretory pathway

PM proteins, lysosome proteins and secretory proteins are first targeted to ER and then to the Golgi apparatus for modifications before being transported elsewhere. In contrast, proteins that are resident in the ER and the Golgi are retrieved and retained in these two organelles. For example, the TMPRSS3 protein (transmembrane protease serine 3), which mutation results in autosomal recessive neurosensory deafness was found to localize exclusively in the ER (Fig. 2), as described [28].

For some proteins, the entire protein-processing pathway could be traced throughout different organelles. Good examples are two integral PM proteins that are encoded by the *KCNE1 *(Fig. 2) and *KCNE2 *genes, both of which belong to the potassium channel, voltage-gated, ISK-related subfamily. Figure 3A shows consecutive steps of KCNE2 protein maturation, with its final destination being the PM, where it acts as a K2+ channel. Here, we report for the first time, that these proteins localize also to the lysosomes. This co-localization of KCNE2 protein with the lysosome marker LAMP2 (lysosomal-associated membrane glycoprotein 2) (Fig. 3A) could reflect the ability of the PM proteins to shuttle between the PM and the endosome/lysosome compartment. Alternatively, the lysosomal location of KCNE1 and KCNE2 proteins could result from their direct transport from the Golgi to the lysosome. It is not clear which of the two mechanisms accounts for this dual localization pattern. Interestingly, the KCNJ6 potassium channel protein, which is a member of subfamily J, never showed the lysosomal resident pattern and localized at the PM only in the same cell array experiment (Fig. 2).

### Cell cycle-dependent protein translocation

In the case of the BACH1 and CHAF1B proteins, we observed a cell cycle-dependent localization pattern. Regarding the BACH1 protein, we observed a complex cell division-dependent localization pattern. At interphase, BACH1 was distributed in the entire cytosol. During the early phase of mitosis, however, the protein accumulated in the vicinity of condensing chromatin (Fig. 3B). The chromatin assembly factor I p60 subunit (CHAF1B) protein translocated from the nucleus into the cytoplasm during cell division (Fig. 3B). As reported [29], we observed most of the CHAF1B protein localizing in the nucleus at interphase, where it is involved in chromatin assembly and DNA replication. After cytokinesis had occurred, we detected the presence of the CHAF1B protein in the cytoplasm of two daughter cells, probably in its inactive form after dissociation from chromatin.

### The effects of protein over-expression on cell morphology

Clusters of cells transfected with gene *CLDN8 *or *CLDN14 *were found to undergo morphological changes. Overexpression of fifty of the 52 proteins was not associated with any abnormal cell morphology.

**Figure 4:**
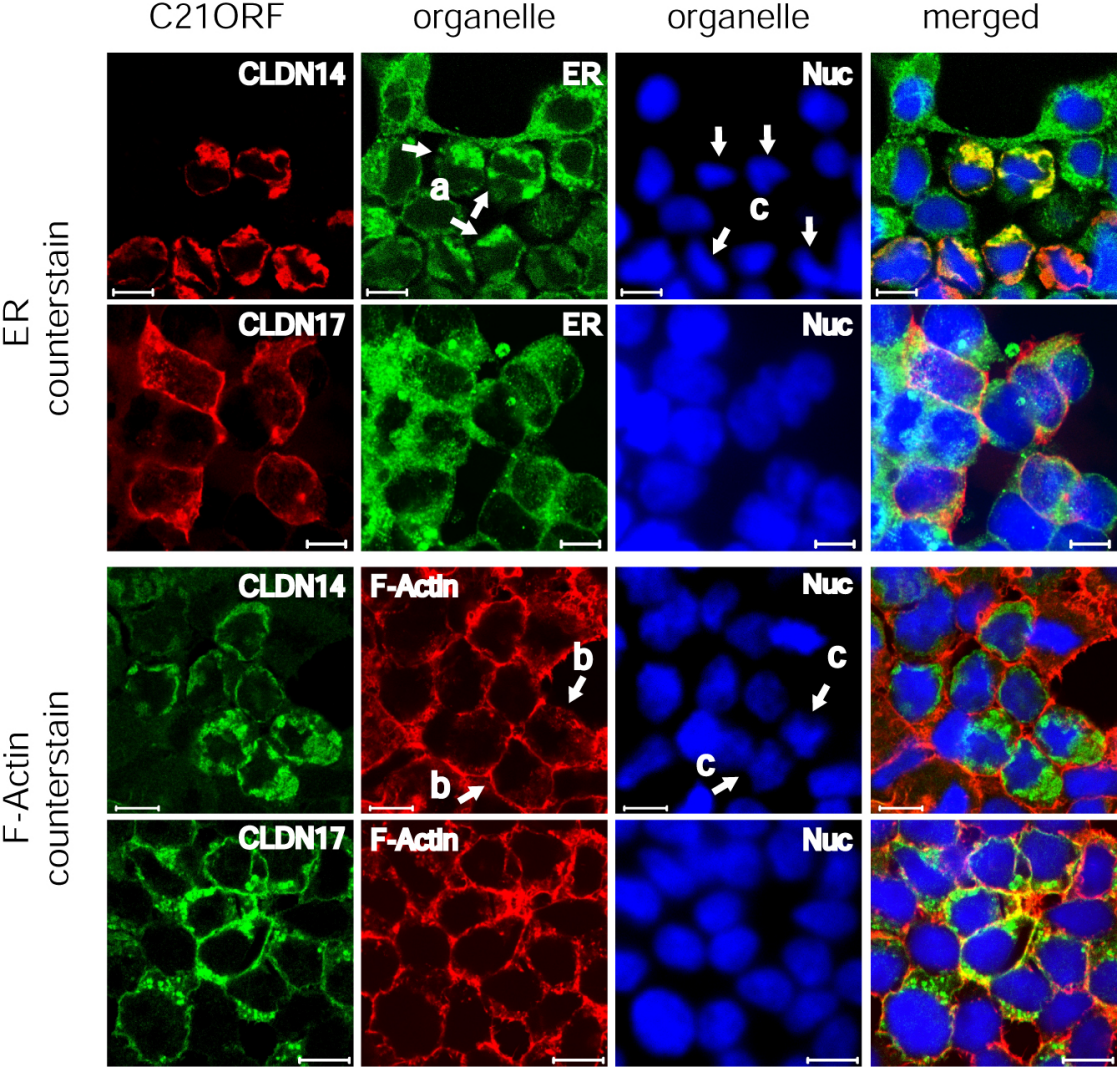
Influence of Chr21 protein over-expression on cellular physiology. Over-expressed claudin-14 was retained in the retracted endoplasmic reticulum (a), which resulted in loss of cell-membrane protrusions (b). Claudin-17 localized in the plasma membrane and its expression did not influence cell morphology. A distinct change in nuclear shape (c) was observed in cells expressing claudin-14 but not in those expressing claudin-17. The scale bar represents 10 μm.

Claudins comprise one of the two major integral membrane protein families that are found in the tight junctions of epithelial cells and endothelial cell sheets. There are more than 20 members of the human claudin gene family, which are characterized by heterogeneous tissue localization patterns [30,31]. Daugherty *et al*. found that translocation of claudin-5 from the PM to an intracellular compartment helps to regulate tight junction permeability during the differentiation of human fetal lung alveolar epithelial cells to a type II cell phenotype [32]. Loss of human claudin-14 results in autosomal recessive deafness [33]. Furthermore, loss of claudin-3 may initiate cilia degeneration of the retinal pigment epithelium [34] and correlates with experimental autoimmune encephalomyelitis [35]. Despite the clinical relevance of the claudin proteins, their structure within tight junctions and the regulatory mechanisms of protein expression is not entirely understood. Here, we report for the first time subcellular localization data for claudin-17, which localizes predominantly at the PM (Fig. 4). The localization of claudin-14 has been described recently [36]. We observed several morphological changes of HEK293 cells as a result of over-expression of these proteins. Expression of claudin-8 and claudin-14, but not claudin-17, resulted in loss of cell-membrane protrusions and cell retraction. Moreover, a distinct change in nuclear shape could be observed in cells expressing claudin-8 (not shown) and claudin-14, but not in those expressing claudin-17 (Fig. 4).

**Figure 5:**
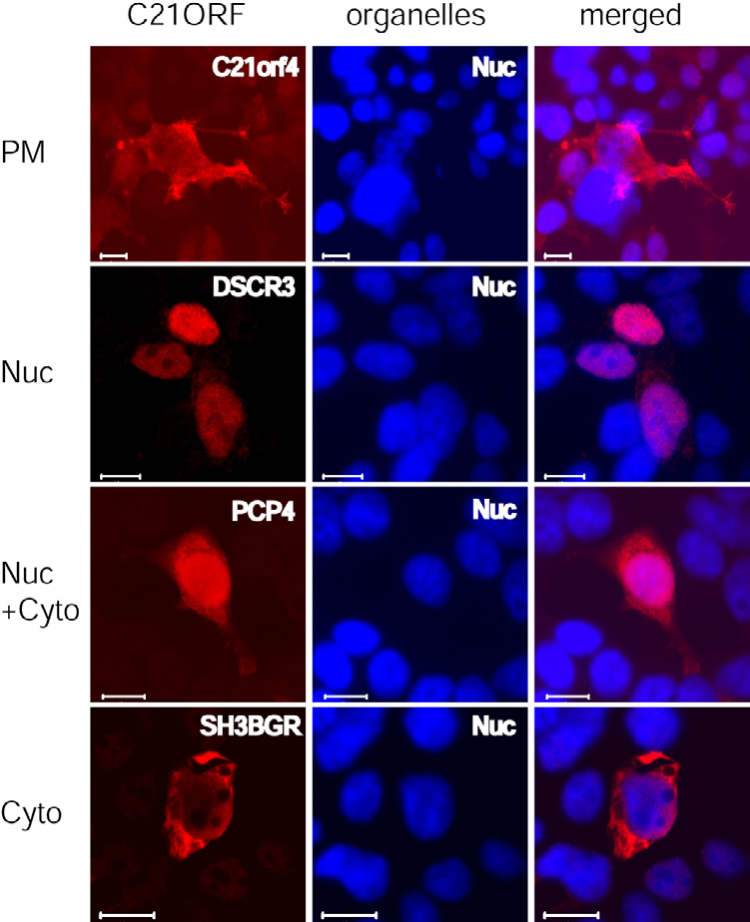
Subcellular localization of proteins of possible relevance to Down's syndrome pathogenesis. The localization pattern of 21 Chr21 proteins related to Down's syndrome critical region was described in this study. The figure shows localization pattern for four of these proteins. Gene names are shown in the left panel, together with an indication of the protein localization. The middle panel shows nucleus DAPI staining. The scale bar represents 10 μm.

Subcellular localization of 34 Chr21 proteins is reported here for the first time. Figure 5 shows the localization patterns of four selected proteins that are encoded by the *C21orf4*, *DSCR3*, *PCP4 *and *SH3BGR *genes [see Additional file 1]. The C21orf4 protein was predicted to be an integral membrane protein. Indeed, we determined its localization in the PM. The *DSCR3 *gene has significant homology to the *Hbeta58 *mouse gene, which is active in embryogenesis [37]. In our study, the DSCR3 protein was found in the nucleus, suggesting that it functions in the regulation of transcription or in the maintenance of nuclear structure. The *PCP4 *gene encodes brain-specific polypeptide PEP-19, which has a key role in brain development [38]. A precise function of the PEP-19 in this process has not, however, been determined. Here, we report localization of the PEP-19 in the cytosol and in the nucleus (Fig. 5).

In Chr21 trisomic cells, genes are usually over-expressed by 1.5-fold in comparison to diploid cells. There are, however, some exceptions, as described recently [11]. The *SH3BGR *and *KCNE2 *genes showed a 2.61-fold and 3.39-fold increase of expression in the kidney and midbrain, respectively, in the Down's syndrome mouse model. In our study, the SH3BGR protein revealed a uniform localization pattern in the cytosol (Fig. 5). As described above, the KCNE2 protein showed a variable distribution pattern, probably due to the differential level of expression in particular cells (Fig. 3A). The abundance of the KCNE2 protein in the PM was lower than that in the lysosome/endosome (Fig. 3A). This abundance-dependent protein localization pattern might be particularly relevant in the case of Chr21 trisomic cells, which over-express the *KCNE2 *gene. This hypothesis, however, needs further functional evaluation.

## Conclusion

This study provides comprehensive information on the subcellular distribution of human Chr21 proteins and the physiological effects following over-expression for some of these genes. This cell phenotype-based information should contribute to further understanding of the molecular pathology of Down's syndrome. The study has not been performed in human Chr21 trisomic cells due to difficulties in reverse transfection of primary cells or immortalized primary cells. Nevertheless, the data provided in this report may serve as a reference for comparative studies concerning aberrations of protein localization as a result of trisomy 21. Cellular localization of many Chr21 proteins is described here for the first time. Together with gene expression profiling and *in situ *hybridization data, knowledge about the protein compartmentalization pattern should contribute to creation of an integrated picture of Chr21 molecular biology. Furthermore, the large-scale localization data obtained in this study support the concept of application of array-based gene expression systems for the evaluation of protein functions on a single-cell level. It shows a versatility of the cell array technology for analysis of gene sets at various levels of genetic information flow. For example, cell arrays allow for parallel gene silencing [39] or cellular localization studies of large gene sets, resulting in cost-effective characterization of protein functions on a genomic scale.

The cell array technology applied in this study has some limitations. Currently, only adhesive cells growing as a monolayer can be used for reverse transfection. Moreover, efficient transfection of primary cells such as fibroblasts or macrophages remains a substantial obstacle. Recently published lentivirus-based "reverse transduction" of the cells could dramatically improve DNA delivery into those difficult-to-transfect cells [40]. Taking advantage of wide cellular tropism of the lentiviruses, a variety of cell types could be reverse transfected using a single vector construct.

Regarding therapeutic purposes, a cell array-based high-throughput localization platform might facilitate the functional screening of chemical compounds that interfere with the expression and translocation of the proteins involved in a particular disease. We believe that cell array-based, high-throughput screening of large genes sets, based on both gene over-expression and knockdown, will accelerate functional analysis of the vast amount of sequence information that has emerged as a result of genome sequencing projects.

## Methods

### Construction of Chr21 ORF plasmid

ORF sequences were obtained from the Chr21 gene catalogue [41]. A total of 207 primer pairs were designed using PRIDE software [42] and used to amplify each ORF from cDNA (Human MTC Panels I+II and QUICK-Clone cDNA; Clontech, Heidelberg, Germany) or from public IMAGE or MGC cDNA clones (RZPD, Berlin, Germany). The PCR products were cloned into the pEntry vector pDONR201. We verified the correct identities of the inserts by generation of 5' and 3' ORF sequence tags. In all, 89 cloned ORFs were selected for re-cloning into the Gateway™ mammalian expression vector pDEST26, which carries an amino-terminal His_6 _fusion tag. For the expression of C-terminal tagged proteins, the ORFs were cloned into the Gateway pDEST-474 vector containing a Myc tag at the C-terminus (kind gift from Dr. Esposito, NCI-Frederick Vector Engineering Group). Such prepared clones were sequenced to confirm the presence of the full length of the insert. Organelle-specific control proteins (kdelr1, pmp22, pmp26, lapC1, laminA and tgn38) were also cloned in the pDEST26 vector.

### Microarray preparation

Before spotting, all purified plasmids were diluted in 0.2% (w/v) gelatin solution to reach final concentrations of 50–100 ng/μl of plasmid and 0.18% gelatin. Depending on the number of plasmid samples in each test, manual spotting or robotic spotting (arrayer from Virtek, Toronto, Canada) was applied to print the plasmid DNA onto standard glass slides coated with poly-L-lysine (Sigma, Munich, Germany). The spotting procedure was carried out using split pins (type SMP4, TeleChem, Sunnyvale, CA) in a hood set at 55% humidity. Each spot was 120 μm in diameter, with a distance of 400 μm between adjacent spots. Each DNA sample was spotted in triplicate.

### Cell culture and reverse transfection

Human HEK293T/17 (ATCC, CRL-11268) cells were cultured in DMEM medium (Invitrogen, Karlsruhe, Germany) supplemented with 10% (v/v) fetal calf serum (Biochrom, Berlin, Germany), L-glutamine (Sigma) and gentamicin (Gibco Invitrogen, Karlsruhe, Germany) at 37°C in a humidified 5% CO_2 _incubator. One day before transfection, 10 × 10^6 ^HEK293T cells per 10-cm dish in 10 ml of medium were seeded out. Prior to transfection, the arrayed slides were covered with Hybriwell (Grace Bio-Labs, Bend, OR) and 190 μl of Effectene transfection reagent (Qiagen, Hilden, Germany) was added, as described [3]. After incubation for 25 min, the Hybriwell and transfection reagent were removed and the slides were placed into a QuadriPerm chamber (Greiner, Frickenhausen, Germany). Subsequently, 3.5 × 10^6 ^HEK293T cells were added on top of each slide for reverse transfection. Following transfection for a period of 48–72 hours, cells were fixed and subjected to immunofluorescence staining.

### Immunofluorescence

Recombinant Chr21 proteins were detected with Penta•His Alexa488 or Alexa555 conjugated mouse antibody (Qiagen). For cellular compartment labeling, the mouse antibody against LAMP2 (H4B4 from Developmental Studies Hybridoma Bank, Iowa City, IA) for lysosomes, against PDI for ER (Stressgen, San Diego, CA), against adaptin-γ for the Golgi complex (BD Biosciences, Heidelberg, Germany), against α-tubulin for microtubule (Sigma), against vimentin for intermediate filaments (Affinity BioReagents, Golden, CO), rabbit antibodies against prohibitin for mitochondria and against catalyse for peroxisome (Abcam, Cambridge, UK) were used. Rhodamine-labeled phalloidin (Molecular Probes Invitrogen, Karlsruhe, Germany) and 4',6-diamidino-2-phenylindole (DAPI) (Sigma) were used to stain F-actin and DNA. After fixation with 100% methanol or 3.7% (v/v) paraformaldehyde, the cell array was permeabilized with 0.1% (v/v) Triton X-100 or 0.5% (w/v) saponin and subsequently blocked for 1 hour at room temperature using 5% (w/v) bovine serum albumin or 5% (v/v) normal serum from the host species of the fluophor-labeled antibodies. Cell arrays were incubated with rhodamine-labeled phalloidin or the rabbit primary antibodies together with mouse anti-His antibody. Incubation with secondary antibody was performed with Alexa488-conjugated donkey anti-rabbit IgG antibody (Molecular Probes) or Cy3-conjugated mouse anti-rabbit IgG antibody (Jackson ImmunoResearch, Cambridge, UK). The organelle-specific mouse antibodies were always used before anti-His antibody staining. Secondary staining was performed with Alexa488 or Alexa568 goat anti-mouse IgG antibody (Molecular Probes). After washing with phosphate-buffered saline, the slides were incubated with 20% (v/v) normal mouse serum for 1 hour at room temperature. The fluophor-conjugated anti-His antibody was then added to detect the fusion protein. Each cell array slide was incubated with DAPI before the final washing and mounting with Prolong Gold anti-fade reagent (Molecular Probes).

### Laser scanning and microscopy

To observe Chr21 proteins within each cell cluster, a BioCCD scanning system (Applied Biosystems, Darmstadt, Germany) was used. For cell morphology analysis and protein subcellular localization, ImagerZ1 microscope (Zeiss, Jena, Germany) and LSM510 confocal system (Zeiss) were used, together with Axiovision 4.0 and LSM510 software (Zeiss).

## Abbreviations

Chr21, chromosome 21; ER, endoplasmic reticulum; LAMP2, lysosomal membrane glycoprotein 2; ORF, open reading frame; PDI, protein disulphide isomerase; PM, plasma membrane; TCA, transfected-cell array

## Authors' contributions

Y.H.H. optimized the cell array protocol, performed protein localization studies and was pivotal in writing the manuscript; H.J.W. performed cloning of expression constructs and clone sequencing and contributed to data analysis; D.V. was pivotal in establishment of the transfected-cell array protocol; F.W. was instrumental in optimizations and production of DNA arrays for reverse transfections; A.F. contributed to development of the His_6 _tag detection protocol; S.T. assisted in maintenance of cell cultures; P.K. was involved in the conceptualization; H.L. is the Head of the Department of Vertebrate Genomics at the Max Planck Institute for Molecular Genetics. M.L.Y. and M.J. supervised the study and were involved in the conceptualization and writing.

## Supplementary Material

Additional File 1 112 Kb. Data description: Table 1 Click here for file
