# Supplementary material for: Cell array-based intracellular localization screening reveals novel functional features of human chromosome 21 proteins
Source: BMC Genomics. 2006 Jun 16;7:155. doi: 10.1186/1471-2164-7-155 (PMC1526728; doi:10.1186/1471-2164-7-155)
Supplement: Additional File 1 — 112 Kb. Data description: Table 1 [file 1471-2164-7-155-S1.doc]

| **Table 1. Subcellular localization of 52 human chromosome 21 proteins.** | | | | |  | | | |
| --- | --- | --- | --- | --- | --- | --- | --- | --- |
| **Gene Symbol** | Function class | **Localization in HEK293T^a^** | **Localization in Swiss-Prot** | **RefSeq Acc.No.** | | **OMIM ID^b^** | **GenBank Protein Acc.No.** |  |
| ABCG1 | ATPase | PM/Golgi | ER/Golgi | NM_004915 | | 603076 | CAA62631 |  |
| *AGPAT3** | acyltransferase | ER/PM(less) | __ | NM_020132 | |  | AAH11971 |  |
| *B3GALT5* | galactosyl-transferase | Golgi/ER | Golgi | NM_006057 | | 604066 | NP_006048 |  |
| *BACH1* | transcription regulation | Cyto(punct) Nuc-M-phase | Nuc (predict) | NM_001186 | | 602751 | BAA24932 |  |
| *C21orf103**  *(KRTAP6-1)* | unclear | Cytoplasm | __ | NM_181602 | |  | NP_853633 |  |
| *C21orf19** | unknown | Nuc/Cyto | __ | AF363446 | |  | AAL34462 |  |
| *C21orf25** | unknown | Nuc/Cyto | __ | NM_199050 | |  | XP_032945 |  |
| *C21orf30** | unknown | Nuc | __ | AL117578 | |  | CAB56001 |  |
| *C21orf4** | unknown | PM | __ | NM_006134 | |  | AAC05974 |  |
| *C21orf59** | unknown | Nuc/Cyto | __ | NM_017835 | |  | AAG00496 |  |
| *C21orf69** | unknown | ER | __ | NM_058189 | |  | AAK60445 |  |
| *C21orf7**  *(TAK1L)* | transcription  factor like | Nuc/Cyto | __ | NM_020152 | |  | AAF81754 |  |
| *C21orf96** | unknown | Cyto (punct) | __ | NM_025143 | |  | NP_079419 |  |
| *CBS* | cystathionine-beta-synthase | Cyto | Cyto | NM_000071 | | 236200 ALLELIC VARIANTS / CLINICAL SYNOPSIS | NP_000062 (splicing isoform) |  |
| *CCT8* | chaperonin | Cyto | Cyto | NM_006585 | |  | NP_006576 |  |
| *CHAF1B* | chromatin assembly factor | Nucleoplasm Cyto-M phase | Nuc;  Cyto-M phase | NM_005441 | | 601245 | NP_005432 |  |
| *CLDN14* | tight junction | ER/PM | __ | NM_012130 | | 605608 ALLELIC VARIANTS | AAG60052 |  |
| *CLDN17** | tight junction | PM/Golgi | __ | NM_012131 | |  | CAB60616 |  |
| *CLDN8** | tight junction | ER/PM | __ | NM_012132 | |  | NP_955360 |  |
| *CRYZL1** | oxidoreductase | Cyto | __ | NM_005111 | | 603920 | BAA91605 |  |
| *CXADR* | receptor | PM | Apical junction Basolateral mem. | NM_001338 | | 602621 | NP_001329 |  |
| *DNMT3L** | methyl-  transferase like | Nuc/Cyto | Nuc (predict) | NM_175867 | | 606588 | AAH02560 |  |
| *DSCR3** | unknown | Nuc | __ | NM_006052 | | 605298 | NP_006043 |  |
| *ETS2** | transcription factor | Nuc | Nuc (predict) | NM_005239 | | 164740 | NP_005230 |  |
| *GCFC* (C21orf66)* | transcriptional repressor | cyto | Nuc* (predict) | NM_016631 | |  | AAD34617 |  |
| *HLCS* | protein ligase | Cyto | Cyto/Mito | NM_000411 | | 253270 CLINICAL SYNOPSIS | NP_000402 |  |
| *HMGN1* | DNA binding | Nuc | Nuc | NM_004965 | | 163920 | AAA52676 |  |
| *HSF2BP** | transcription  factor binding | Cyto | __ | NM_007031 | | 604554 | NP_008962 |  |
| *IFNGR2** | receptor | PM/ER | __ | NM_005534 | | 209950 CLINICAL SYNOPSIS | AAH03624 |  |
| *KCNE1** | K-channel | Lyso/PM | __ | NM_000219 | | 176261 ALLELIC VARIANTS / CLINICAL SYNOPSIS | AAH36452 |  |
| *KCNE2** | K-channel | Lyso/PM | __ | NM_172201 | | 603796 ALLELIC VARIANTS | NP_005127 |  |
| *KCNJ15** | K-channel | PM/Golgi | __ | NM_002243 | | 602106 | NP_002234 |  |
| *KCNJ6** | K-channel | PM/Golgi | __ | NM_002240 | | 600877 | NP_002231 |  |
| *KIAA0179* | unknown | Nuc/Cyto(punct) | Nuc; Nucleolus | D80001 | |  | BAA11496 |  |
| *MCM3AP* | DNA binding | Cyto/Nuc | __ | NM_003906 | | 603294 | BAA25170 |  |
| *MxA* | dynamin & large GTPases | Cyto(punct) | Cyto | NM_002462 | | 147150 | NP_002453 |  |
| *NNP-1 (D21S2056E)* | RNA processing | Nucleolus | Nuc; Nucleolus | NM_003683 | |  | AAH00380 |  |
| *PCBP3* | RNA binding | Cyto/Nuc | Cyto | NM_020528 | | 608502 | AAH12061 |  |
| *PCP4** | unknown | Nuc/Cyto | __ | NM_006198 | | 601629 | CAA63724 |  |
| *PDE9A2** | phosphodiesterase | Cyto (accum) | __ | NM_002606 | | 602973 | AAH09047 |  |
| *PDXK* | kinase | Cyto | Cyto | NM_003681 | | 179020 | AAH00123 |  |
| *PFKL** | kinase | Cyto (accum) | __ | NM_002626 | | 171860 | AAH09919 |  |
| *PKNOX1* | transcription factor | Nuc/Cyto | Nuc (predict) | NM_004571 | | 602100 | AAH07746 |  |
| *PPIA3L** | peptidylprolyl isomerase A-like | Nuc/Cyto | __ | XM_351375 | | 123840 | CAA37039 |  |
| *RPS5L** | Ribosomal protein S5-like | Cyto | __ | NG_000903 | |  | pseudogene, 98% identity to RPS5 BAB79493.1 |  |
| *SH3BGR** | SH3 adaptor | Cyto | __ | NM_007341 | | 602230 | AAH06371 |  |
| *TMPRSS3a* | protease | ER | ER | NM_024022 | | 605511 ALLELIC VARIANTS | NP_076927 |  |
| *TSGA2 ** | chromosome-associated | Cyto /Nuc | Cyto/Nuc(metaphase) (predict) | NM_080860 | |  | NP_543136 |  |
| *UBASH3A** | catalytic activity | Cyto | Nuc* (predict) | NM_018961 | | 605736 | NP_061834 |  |
| *UBE2G2** | ubiquitin conjugating enzyme E2G2 | Cyto | __ | NM_003343 | | 603124 | AAC32312 |  |
| *WDR4** | unknown | Nucleoplasm | __ | NM_018669 | | 605924 | AAH06341 |  |
| *WDR9_3'*^c^ (BRWD1)* | unknown | Nuc | __ | NM_033656_5663..6949 | |  | BAD74072 |  |
| Subcellular localization of 52 Chr21 proteins was determined, among which the localization of 34 proteins have been newly reported (indicated by asterisk). In total, the localization patterns were highly consistent with described gene functions. ^a^ Accum: accumulated; Cyto: cytosol; ER: endoplasmic reticulum; Lyso: lysosome and endosome; Mem: membrane; Mito: mitochondrion; Nuc: nucleus; PM: plasma membrane; Punct: punctate; Predict: 3 non-experimental localization qualifiers including “potential”, “probable” and “by similarity”. ^b^ 33 genes had an OMIM entry, among which 7 had known allelic variants associated with diseases, and 4 had a clinical synopsis. ^c^ WDR9_3' stands for 3' fragment of *WDR9* (*BRWD1*) gene containing the nucleotides 5663..6949 of the RefSeq mRNA NM_033656. | | | | | | | | |
